# *Enterobius vermicularis* in Brazil: An integrative review

**DOI:** 10.1590/0037-8682-0073-2023

**Published:** 2023-09-22

**Authors:** Maria Fantinatti, Alda Maria Da-Cruz

**Affiliations:** 1 Universidade Federal de Roraima, Curso de Medicina, Boa Vista, RR, Brasil.; 2 Instituto Oswaldo Cruz, Fundação Oswaldo Cruz, Laboratório Interdisciplinar de Pesquisas Médicas, Rio de Janeiro, RJ, Brasil.; 3 Universidade do Estado do Rio de Janeiro, Faculdade de Ciências Médicas, Disciplina de Parasitologia, Rio de Janeiro, RJ, Brasil.

**Keywords:** Enterobius vermicularis, Prevalence, Frequency, Brazil

## Abstract

*Enterobius vermicularis,* an intestinal helminth, is transmitted through the ingestion of eggs found in food, water, dust, or other fomites, including infected individuals. This review aimed to examine the frequency and distribution of *E. vermicularis* infections in Brazil between 1991 and 2022. The conducted bibliographic survey revealed that the frequency of *E. vermicularis* infections in Brazil ranged from 0.1 to 26.1%, depending on factors such as population ethnicity, individual age group, geographic area, time frame, and diagnostic method. However, these findings were based on a limited number of publications, suggesting that the actual prevalence rates of *E. vermicularis* infection may still be unknown and potentially underestimated.

## INTRODUCTION

Enterobiasis, also known as enterobiosis, is a globally distributed parasitic disease, not confined solely to underdeveloped countries. It is estimated that over 40 million people in the USA are infected with *Enterobius vermicularis*, commonly known as pinworm1^1^ ^,^ ^2^. Notably, a frequent correlation has been reported between enterobiasis and families of lower socioeconomic status^3^.

In Brazil, the proliferation of slums, coupled with the high occupancy rate per dwelling, fosters the spread of parasites, including pinworms. Similarly, settings like day care centers and schools may heighten the risk of disease transmission^3^.

This review aimed to examine the distribution and frequency of *E. vermicularis* infection cases in Brazil from 1991 to 2022.

### METHODOLOGY

An exploratory and descriptive bibliographic review was conducted between January and June 2023, focusing on the prevalence of *E. vermicularis* in Brazil. The research utilized the Medical Literature Analysis and Retrieval System Online (Medline) electronic database and Virtual Health Library (VHL) Regional Portal.

The search for the etiological agent, “*Enterobius*,” was delimited using Medical Subject Headings (MeSH) terms. Additionally, the species’ synonym, “pinworm,” and the geographical area, “Brazil,” were included in the search parameters. The Boolean operators “OR” and “AND” were utilized to combine these descriptors. Consequently, the following search strategy was formulated: 1) ((*Enterobius*) OR (pinworm)) AND (Brazil).

The inclusion criterion for manuscript selection was the full retrieval of articles. Studies were excluded if they were unavailable, utilized archaeological material, did not involve human samples, used samples known to be positive for enteroparasites, did not focus on the *Enterobius* worm, or were bibliographic reviews. In cases of duplicate studies across search platforms, only one was included.

## RESULTS

### Annual number of publications addressing the positivity of *Enterobius vermicularis* in Brazil.

A bibliographic survey of the Medline and VHL electronic libraries yielded 68 and 81 papers, respectively. Following the application of inclusion and exclusion criteria, 56 studies conducted on human samples within the Brazilian territory were selected from Medline and VHL for inclusion in this review (Table 1).

**TABLE 1:** Positivity of *Enterobius vermicularis* in Brazil up to 2022, according year in wich the samples were obtained, city and state, population studied, age, number of individuals or samples, diagnostic technique, prevalence and reference.

Year in which the samples were obtained	City/State	Population studied	Age	Number of individuals or samples	Diagnostic technique	Positivity	Reference
**1984**	Guaporé, Laje e Ribeirão, Pacaas-Novos/RO	Indigenous	All	639	Spontaneous sedimentation	2%	Santos et al. 1985
**1984**	Guarulhos/SP	Schoolers	6-16 years	913	Spontaneous sedimentation	1.9% (6-10 years = 2.6%	Chieffi et al. 1988
						11-16 years = 1%)	
**1987**	Parque Xingu/MT	Indigenous	All	69	Flotation in Sodium Chloride	26.1%	Ferreira et al. 1991
					Spontaneous sedimentation		
**1986-1990**	Campinas/SP	Health center users	All	770	NR	1.4% (1-7 years = 6%)	Gioia 1992
						(8-18 years = 0,9%)	
						(over 19 years = 0%)	
**1992**	Campinas/SP	Farm dwellers	All	82	Centrifuge-sedimentation in formaldehyde-ether	1.4% (under 16 years old = 3.7%)	Kobayashi et al. 1995
						(over 16 years old = 0 %)	
**1989-1990**	Uberlândia/MG	Food handlers	20-66 years	264	Spontaneous sedimentation	1%	De Rezende et al. 1997
**1994**	Uberlândia/MG	Children	4 months-7 years	300	Spontaneous sedimentation	4%	Machado et al. 1998
**NR**	Sorocaba/SP	Preschoolers	Up to 5 years	1.050	Spontaneous sedimentation	1.2%	Coelho et al. 1999
**1992-1996**	São José da Bela Vista/SP	Public hospital users	0-68 years	1.032	Spontaneous sedimentation	2.2%	Tavares-Dias and Grandini 1999
**NR**	Niterói/RJ	Preschoolers and daycare workers	1-6 years	340 (children)	Spontaneous sedimentation	0.8% (children)	Uchôa et al. 2001
			Over 18 years	54 (staff)	Flotation in Zinc Sulfate	0% (staff)	
**NR**	Campinas/SP	Schoolers	6-11 years	146	Spontaneous sedimentation	5%	Iñiguez et al. 2002
					Flotation in Sodium Chloride		
					Flotation in Zinc Sulfate		
					Centrifuge-sedimentation in formaldehyde-ether		
**NR**	Cascavel/CE	General population	All	251	Spontaneous sedimentation	2.4%	Heukelbach et al. 2004
**2001**	Presidente Prudente/SP	NR	1-12 years	1.000	Spontaneous sedimentation	1.9%	Tashima and Simões 2004
					Flotation in Zinc Sulfate		
					Adhesive tape (when requested)		
**2001-2002**	Doutor Camargo,	Rural population	All	181	Spontaneous sedimentation	8.8%	Guilherme et al. 2004
	Ivatuba, Floresta/PR			(32- Floresta	Flotation in Zinc Sulfate	(Floresta = 31.4%	
				107- Ivatuva		Ivatuva = 6.5%	
				42- Doutor Camargo)		Doutor Camargo = 5.2%)	
**2003-2004**	Eirunepé/AM	NR	2 months-80 years	413	Spontaneous sedimentation	0.5%	Araújo et al 2005
**2002**	Botucatu/SP	Preschoolers	0-6 years	279	Spontaneous sedimentation	10.0%	De Carvalho et al. 2006
					Flotation in Zinc Sulfate		
					Centrifuge-sedimentation in formaldehyde-ether		
					Adhesive tape		
**2002-2003**	Uruguaiana/RS	Preschoolers	5 months-6 years	1288	Spontaneous sedimentation	0.8%	Chavez et al. 2006
**2005-2006**	Rio de Janeiro/RJ	Pediatric patients with gastroenteritis	0-5 years	213	Centrifuge-sedimentation in formaldehyde-ether	0.5%	Carvalho-Costa et al. 2007
**1995-2005**	Botucatu/SP	Patients with appendicitis	All	1.600	Histopathological	1.4%	Da Silva et al. 2007
**2005**	Vespasiano/MG	Preschoolers	1-5 years	176	Centrifuge-sedimentation in formaldehyde-ether	2.5%	Barçante et al. 2008
**1969-2004**	Caxias do Sul/RS	Schoolers	6-14 years	9.789	Spontaneous sedimentation (1969-1970)	8%	Basso et al. 2008
					Centrifuge-sedimentation in formaldehyde-ether (1971-2004)		
					Adhesive tape (1980-1982)		
**1996**	Abadia dos Dourados/MG	Rural and Urban population	All	376	Spontaneous sedimentation	0.5% (Rural population =	Machado et al. 2008
						1.1% Urban population = 0%)	
**1996-1997**	Uberlândia/MG	NR	0-15 years	160	Spontaneous sedimentation	8.8%	Machado et al. 2008
**2000**	São Paulo/SP	Children	2-14 years	120	Kato-katz Spontaneous sedimentation	1.7%	Korkes et al. 2009
					Flotation in Zinc Sulfate		
**2004**	Presidente Bernardes/SP	Children	0-6 years	101	Spontaneous sedimentation	8.9%	Tashima et al. 2009
					Flotation in Zinc Sulfate		
**2006**	Berilo/MG	Rural and Urban population	0-90 years	149	Kato-katz	2%	Martins et al. 2009
					Centrifuge-sedimentation in formaldehyde-ether	(Rural population = 1.6%	
						Urban population = 2.3%)	
**2007-2008**	Coari/AM	Preschoolers	NR	211	Spontaneous sedimentation	2.4%	Monteiro et al. 2009
**NR**	Patos de Minas/MG	Children	0-6 years	161	Spontaneous sedimentation	0.6%	Silva and Gonçalves da Silva 2010
**2007-2008**	Chapadinha/MA	General population	All	3933	Spontaneous sedimentation	1%	Silva et al. 2010
**2006-2008**	Porto Alegre/RS	People with special needs	2-60 years	146	Spontaneous sedimentation	3.4%	Silva et al. 2010
					Flotation in Zinc Sulfate		
**2007**	Manaus/AM	General population	0-85 years	400	Spontaneous sedimentation	11%	Oliveira et al. 2010
**2008**	Santa Isabel do Rio Negro/AM	NR	0-5 years	113 (Adhesive tape)	Adhesive tape	15% (Adhesive tape)	Valverde et al. 2011
				463 (Centrifuge-sedimentation in formaldehyde-ether)	Centrifuge-sedimentation in formaldehyde-ether	0.6% (Centrifuge-sedimentation in formaldehyde-ether)	
**NR**	Natal/RN	Children residing in orphanage	4-12 years	86	Adhesive tape	72.1%	Campos et al. 2011
**1996-1997**	Uberlândia/MG	Preschoolers and daycare workers	All	180	Spontaneous sedimentation	2.2%	Machado et al. 2010
**2011-2013**	Parnaíba/PI	Food handlers	20-59 years	251	Spontaneous sedimentation	13%	Fernandes et al. 2014
					Flotation in Sodium Chloride		
**N**R	Miranda/MS	Indigenous	1-33 years	134	Spontaneous sedimentation	3%	Neres-Norberg et al. 2014
					Flotation in Sodium Chloride		
**2010**	Pelotas/RS	Ostomized individuals	All	71	Centrifuge-sedimentation in formaldehyde-ether	2.8%	Santos et al. 2014
					Flotation in Zinc Sulfate		
**2012**	Florianópolis/SC	Preschoolers	2-6 years	57	Spontaneous sedimentation	1.8%	Santos et al. 2014
					Flotation in Zinc Sulfate		
**2015**	Londrina/PR	General population	Over 18 years	187	Spontaneous sedimentation	0.5%	Benitez et al. 2016
					Flotation in Zinc		
					Sulfate		
					Flotation in Sodium Chloride		
**2011-2014**	Caxias do Sul/RS	Food handlers	All	331	NR	0.3%	Porto et al. 2016
**2010-2011**	Ituiutaba/SP	Preschoolers and daycare workers	0-10 years	181 (children = 140	Spontaneous sedimentation	2.3% (children)	Moura et al. 2017
			Over 18 years	Staff = 41)	Centrifuge-sedimentation in formaldehyde-ether	2.4% (staff)	
					Flotation in Sodium Chloride		
**2012-2013**	Ribeirão Preto/SP	NR	3-12 years	576	TF-Test Kit	7.3%	Fonseca et al. 2017
**NR**	São Matheus/ES	intellectual and/or multiple deficiency	15-61 years	50	Spontaneous sedimentation	10.7%	Oliveira Albuquerque and Andrade de Souza 2017
**NR**	Duque de Caxias/RJ	NR	1-85 years	180	Spontaneous sedimentation	13%	Valença Barbosa et al. 2017
**2015**	Santo Antonio de Jesus/BA	Rural population	All	144	Spontaneous sedimentation	10.4%	Andrade et al. 2018
					Adhesive tape Kato-katz		
**2013**	Sumidouro/RJ	General population	2-87 years	294	Spontaneous sedimentation	0.7%	Barbosa et al. 2018
					Flotation in Sodium Chloride		
**2016**	Ipê/RS	Rural and Urban population	6-11 years	124	Spontaneous sedimentation	20%	Zanotto et al. 2018
					Flotation in Zinc Sulfate		
**2008-2009 (Viçosa)**	Viçosa e Muriaé/MG	Rural and Urban population	All	419 (Viçosa)	Spontaneous sedimentation	0.95% (Viçosa)	Iasbik et al. 2018
**2007-2015 (Muriaé)**				1832 (Muriaé)		0.05% (Muriaé)	
**2016**	João Pessoa/PB	Schoolers	5-16 years	150	Spontaneous sedimentation	5.7%	Monteiro et al. 2018
					Para-test kit		
**2013**	Diamantina/MG	Quilombola community	All	78	Spontaneous sedimentation	0.0%	Eustachio et al. 2019
**2018**	Bandeirantes/PR	Schoolers	10-15 years	112	Spontaneous sedimentation	0.9%	Almeida et al. 2020
					Flotation in Zinc		
					Sulfate		
					Kato-katz		
**2015**	Maceió/AL	General population	All	1.581	Spontaneous sedimentation	0.4%	Araújo et al. 2020
**2017-2019**	Vitória da Conquista/BA	Children and adolescents attending non-governmental organizations	4-17 years	116	Spontaneous sedimentation	2.6%	Alves et al. 2021
**2016**	Foz do Iguaçu/PR	Preschoolers	3-5 years	178	Spontaneous sedimentation	1.7%	Ferreira et al. 2021
					Flotation in Zinc Sulfate		
**2015-2016**	Sinop/MT	Preschoolers and Schoolers	3-12 years	646	Spontaneous sedimentation	0.8%	Carvalho et al. 2022
					Flotation in Zinc		
					Sulfate		
					Flotation in Sodium Chloride		
**2017-2019**	Rio Branco/AC	General population	All	53.199	Spontaneous sedimentation	0.1%	Sinhorin et al. 2022

NR: Not reported.

The research conducted for this review did not specify a time frame concerning the initial published study. The earliest three studies identified originated from the 1960s (two in 1965 and one in 1967); however, owing to their unavailability, they were excluded from this review. Consequently, the first study incorporated into this review dates back to 1985, involving a survey conducted using clinical stool samples from the indigenous population of Pacaas-Novos in the state of Rondônia^4^.

*E. vermicularis* has been detected in human feces across all regions of Brazil. However, a greater number of studies (28/56)^3^ ^,^ ^5^ ^-^ ^31^ originate from the Southeast region. Despite this region boasting the highest human development indices in the country, and thus an anticipated lower frequency of enteroparasite infection, it also houses the majority of research institutes/universities and receives the most science-related investments. This fact may account for our findings.

In 19 out of 27 states, including the Federal District, there were no reported instances of *E. vermicularis* circulation. This absence of reports could be attributed to inadequate research methods for the parasite, insufficient investment in research, or less plausibly, the absence of helminths in these states.

### Diagnostic strategies for *Enterobius vermicularis*

The available data on the frequency of *E. vermicularis* was derived from 25 studies that employed more than one technique, each based on different methodological principles^3^ ^,^ ^12^ ^-^ ^14^ ^,^ ^20^ ^-^ ^22^ ^,^ ^25^ ^,^ ^29^ ^,^ ^32^ ^-^ ^47^. Conversely, 29 studies utilized a single technique, either in isolation or in conjunction with helminth larvae concentration techniques^4^ ^,^ ^5^ ^,^ ^7^ ^-^ ^11^ ^,^ ^15^ ^-^ ^19^ ^,^ ^23^ ^,^ ^24^ ^,^ ^26^ ^-^ ^28^ ^,^ ^30^ ^,^ ^31^ ^,^ ^35^ ^,^ ^48^ ^-^ ^56^. Of these, sedimentation methods were the most frequently employed (23/29)^4^ ^,^ ^5^ ^,^ ^8^ ^-^ ^11^ ^,^ ^18^ ^,^ ^19^ ^,^ ^23^ ^,^ ^24^ ^,^ ^27^ ^,^ ^28^ ^,^ ^30^ ^,^ ^31^ ^,^ ^35^ ^,^ ^48^ ^-^ ^52^ ^,^ ^54^ ^-^ ^56^.

This review found that only 6 out of 56 studies utilized adhesive tape for diagnosis^3^ ^,^ ^14^ ^,^ ^34^ ^,^ ^36^ ^,^ ^42^ ^,^ ^53^. The adhesive tape method demonstrated higher sensitivity in diagnosing *E. vermicularis* infection^42^ compared to other methods. In one study, the gummed tape method was employed for a shorter duration than the entire study^34^, while in another, the method was only applied when clinically indicated^14^. Given the biological characteristics of *Enterobius*, the Graham method could potentially enhance positivity in studies. The Graham method has also been employed for environmental samples, such as public restroom surfaces and transportation means, where *Enterobius* presence has been reported in Brazil^10^ ^,^ ^57^ ^-^ ^59^.

The methodologies employed in these studies, either individually or in combination, indicate a broad investigation into the presence of protozoa and geohelminths. Occasionally, evolutionary forms of *E. vermicularis* were observed. This observation underscores the apparent lack of interest in *E. vermicularis* research. Consequently, most of the existing data on the frequency of *E. vermicularis* infections are derived from studies with experimental designs that are not adequately suited for research on this particular helminth.

### Prevalence of *Enterobius vermicularis* in Brazil

In Brazil, *E. vermicularis* notification is not mandatory, and no nationwide study exists to investigate this helminth. Consequently, determining the prevalence of *E. vermicularis* is challenging owing to the reliance on data from independent, cross-sectional research conducted by various groups with local scope and specific populations. Furthermore, the lack of standardization in diagnostic methodologies complicates this task. The highest frequency of *E. vermicularis* was found in samples collected in 1987 from the indigenous population of Xingu Park in Mato Grosso, with a prevalence of 26.1%. These samples were analyzed using sedimentation in water and sodium chloride solution floating techniques^32^. Despite employing similar diagnostic methods, other studies conducted in Rondônia and Mato Grosso do Sul on samples from indigenous populations reported a significantly lower frequency of *E. vermicularis* (2% and 3%, respectively)^4^ ^,^ ^38^.

Only a single study evaluated individuals with special needs, rendering it impossible to ascertain the frequency within this population in the country^60^.

*E. vermicularis* infection predominantly impacts children^5^ ^,^ ^6^ ^,^ ^7^ ^,^ ^12^ ^,^ ^24^ ^,^ ^56^. Excluding the study conducted in Sumidouro/RJ and Ituiutaba/SP^25^ ^,^ ^29^, the infection frequency among children-only studies ranged from 0.5% in Rio de Janeiro/RJ to 72.1% in Natal/RN. The infection rates among children in Brazil fluctuate based on the region under study and the characteristics of the child population, such as family income, residential area (rural or urban), and primarily, their exposure to crowded environments. The presence of *Enterobius* in consumable vegetables has also been documented^20^ ^,^ ^61^, suggesting these foods may serve as a transmission route for helminths in Brazil. Among food handlers, the infection frequency varied from 0.3 to 13% depending on the region^8^ ^,^ ^37^ ^,^ ^62^, underscoring the potential role of this population in *Enterobius* transmission, as highlighted in the study by Fernandes et al. (2014)^37^.

The presence of infective *E. vermicularis* eggs in public environments, such as bathrooms and buses, is concerning because of the possibility of contamination^10^ ^,^ ^57^ ^-^ ^59^. This potential for contamination elucidates the sporadic instances of *E. vermicularis* infection.

Anal pruritus was a common complaint (50%) among children attending a daycare center in a Rio de Janeiro/RJ community. This prompted an investigation into the prevalence of *E. vermicularis* infection within this demographic. In 2015, 5.9% (4/68) of the children, aged 1-4 years, were found to be infected with *E. vermicularis*, as determined by the Graham method.

Over the past 6 months, drugs were administered to more than 22% of these children to treat worms infection. The reported usage of antiparasitic drugs, in conjunction with the frequency of enteroparasite occurrence and its primary clinical manifestation, prompted us to question the estimated prevalence rates of helminths in Brazil.

The papers reviewed generally indicated that data on *E. vermicularis* infection frequency are derived from studies primarily designed to survey intestinal parasites. Therefore, the diagnostic methodologies employed exhibited lower sensitivity in detecting parasite eggs. An exception was the study by Campos et al. (2011), which aimed to ascertain pinworm prevalence and its association with enuresis cases in children from an orphanage^53^. The high infection frequency observed in this study (72.1%) resulted from three factors: 1) the age range of the population; 2) overcrowding; and 3) the diagnostic methodology, inclusive of sample collection preparation.

## FINAL CONSIDERATIONS

The prevalence of *E. vermicularis* infections in Brazil between 1991 and 2022 ranged from 0.1-72.1%. This variation was influenced by factors such as population ethnicity, age group, geographic location, time period, and diagnostic methods. However, these statistics were derived from a limited number of publications, suggesting a potential underestimation of the actual prevalence rates of *E. vermicularis* infection.

*E. vermicularis* eggs, already infective upon release, can be transmitted directly through person-to-person contact. This makes crowded environments such as day care centers, schools, and nursing homes conducive to the spread of this helminth. Furthermore, in Brazil, favelas are home to over 17 million people, constituting 8% of the population^63^. In these settings, population agglomeration in intra- and peridomiciles is common, thereby promoting (re)infection. In such high-frequency environments, recurrent infections are anticipated. Consequently, the chronicity of *E. vermicularis* infection may be more prevalent than anticipated, and the issue of parasite resistance to available drugs is a subject of ongoing debate.

In Brazil, many states and municipalities lack reports of *E. vermicularis* infection, while others present only few reports, indicating that the geographical distribution of this infection requires further investigation. The majority of these studies are typically cross-sectional, stemming from individual research groups, and primarily focus on local parasitological surveys. However, the methodologies employed are often incomparable and may not be the most sensitive for parasite identification. The application of unsuitable techniques can result in false-negative outcomes, potentially extending the parasite-host interaction, which may cause perianal itching and gastrointestinal symptoms. Additionally, the infection has been linked to conditions such as vulvovaginitis and appendicitis.

This review consolidates studies published from 1985 to 2022, showcasing the prevalence of enterobiasis across various Brazilian states and the diagnostic strategies employed. In 13% (7/54) of these studies, an *E. vermicularis* frequency equal to or exceeding 10% was noted. However, the absence of a national infection survey and a dearth of research targeting parasite identification precludes definitive conclusions about whether the distribution of *E. vermicularis* is on the rise or decline. The limited understanding of *E. vermicularis* transmission dynamics hampers the development of strategic control measures targeting potential transmission sources.
